# Enhancement in Electromagnetic Wave Shielding Effectiveness through the Formation of Carbon Nanofiber Hybrids on Carbon-Based Nonwoven Fabrics

**DOI:** 10.3390/nano11112910

**Published:** 2021-10-30

**Authors:** Hyun-Ji Kim, Gi-Hwan Kang, Sung-Hoon Kim, Sangmoon Park

**Affiliations:** Department of Energy and Chemical Engineering, Silla University, Busan 46958, Korea; khyunj32@gmail.com (H.-J.K.); nice_gyan@naver.com (G.-H.K.); spark@silla.ac.kr (S.P.)

**Keywords:** carbon nanofiber hybrids, nonwoven fabrics, hydrogen incorporation, selective formation, cyclic process, nonselective formation, electromagnetic wave shielding effectiveness

## Abstract

The selective hybrid formation of numerous tiny carbon nanofibers (CNFs) in carbon-based nonwoven fabrics (c-NFs), namely CNFs formed only on the surfaces of individual carbon fibers (i-CFs) constituting c-NFs and not on the surfaces of carbon microcoils (CMCs), could be formed by the incorporation of H_2_ gas flow into the C_2_H_2_ + SF_6_ gas flow in a thermal chemical vapor deposition system. On the other hand, the nonselective hybrid formation of numerous tiny CNFs in c-NFs, that is, tiny CNFs formed on the surfaces of both i-CFs and CMCs, could be achieved by simply modulating the SF_6_ gas flow on and off in continuous cycles during the reaction. Detailed mechanisms are suggested for the selective or nonselective formation of tiny CNFs in c-NFs. Furthermore, the electromagnetic wave shielding effectiveness (SE) values of the samples were investigated across operating frequencies in the 8.0–12.0 GHz range. Compared with previously reported total SE values, the presently measured values rank in the top tier. Although hybrid formation reduced the electrical conductivity of the native c-NFs, the total SE values of the native c-NFs greatly increased following hybrid formation. This dramatic improvement in the total SE values is ascribed to the increased thickness of c-NFs after hybrid formation and the electromagnetic wave absorption enhancement caused by the intrinsic characteristics of CMCs and the numerous intersections of tiny CNFs.

## 1. Introduction

Electromagnetic (EM) wave radiation emitted from electronic devices operating at high frequencies can interfere with the accurate function of other electronics. Therefore, electromagnetic wave interference (EMI) shielding of both electronics and radiation sources is required to prevent the malfunction of electronic devices. EM waves are composed of oscillating electric and magnetic fields. Therefore, materials with EM wave shielding capabilities are expected to interact with either one or both of these fields. For an efficient absorption loss greater than 10 dB, reflection and absorption loss are regarded as the main shielding mechanisms among the three major shielding routes (reflection, absorption, and multiple reflection) [1,2,3,4]. In the relatively low frequency range (typically less than 2.0 GHz), conductive metals are considered to be appropriate materials for EMI shielding via the reflection loss route [4,5]. At high operating frequencies (above 2.0 GHz), which are required by more than five generations of mobile communications, the absorption loss route of shielding materials is a crucial mechanism for preventing EMI [4,5]. The electrical conductivity is the key parameter for the reflection loss mechanism, while the absorption loss mechanism requires both electrical conductivity and magnetic permeability [1,5]. Therefore, materials with EM wave shielding capabilities at high operating frequencies should have superior magnetic characteristics and high electrical conductivity to optimize the absorption loss mechanism.

Compared with metals, carbon-based materials can have flexibility, light weight, and high magnetic field characteristics. In these respects, carbon-based nonwoven fabrics (c-NFs) are promising EMI shielding materials for high operating frequencies [6,7]. c-NFs consist of randomly oriented carbon fibers. Consequently, when an incoming EM wave reaches these fibers, electric current flows through randomly oriented carbon nanofibers (CNFs) in various directions, thereby inducing an electromotive force and generating a variable magnetic field [8,9]. The geometry of c-NFs holds and rotates incoming EM waves through the generated variable magnetic field. Thus, incoming EM wave energy is absorbed into c-NFs and is finally converted into thermal energy [10]. Accordingly, c-NFs can efficiently absorb EM waves, thus shielding against such waves.

Recently, hybrid formation reactions on carbon-based materials have been conducted to enhance shielding effectiveness (SE) values [6,11,12,13]. Indeed, the formation of carbon fiber–carbon microcoil (CF–CMC) hybrid materials on c-NFs was reported in our laboratory [6]. Despite the reduction in electrical conductivity, the SE value of CF–CMC hybridized nonwoven fabrics was above 60 dB throughout the entire range of operating frequencies, from 8.0 to 12.0 GHz. Kang et al. also reported that composites comprising carbon nanocoil–carbon microcoil hybrids in polyurethane showed higher shielding effectiveness than composites comprising carbon microcoils (CMCs) in polyurethane in the operating frequency range 1.25–4.0 GHz, irrespective of the mixing ratio of carbon nanomaterials in polyurethane [11]. By extension, Gamage et al. reported that a multi-walled carbon nanotube (MWCNT)-coated free-standing carbon fiber (MWCNT/CF) fabric showed a high SE in the operating frequency range of 0–3 GHz [12]. Kong et al. reported that a porous carbon nanotube/reduced graphene oxide (CNT/RGO) composite had powerful absorbing SE in the operating frequency range of 8.2–12.4 GHz [13]. These results indicate that hybrid formation can enhance SE values.

In the present work, we investigated the appropriate materials and processes for the hybrid formation reaction and the shielding mechanism by which hybrid formation enhances SE values. Hybrids comprising tiny CNFs on the CMCs and/or individual carbon fibers (i-CFs) in c-NFs were fabricated. In particular, the selectivity of the hybrid formation of tiny CNFs was controlled by incorporating hydrogen gas flow and introducing a cyclic process. The morphologies, the elemental compositions, crystal structures, and operating frequency-dependent SE values of the CNF-hybrids in c-NFs were investigated. Based on the results, detailed mechanisms of the selective or nonselective hybrid formation of tiny CNFs and the main shielding mechanism of the formed hybrids are suggested and discussed.

## 2. Materials and Methods

As a catalyst for the formation of tiny CNF-hybrids on c-NFs, approximately 0.01 g of bunch-type Ni powder (99.7%), with particle diameters ranging from 100 to 200 μm, was spread onto the prepared c-NFs on a 2 mm thick alumina substrate. A thermal chemical vapor deposition (TCVD) system was employed for the formation of tiny CNF-hybrids on c-NFs using C_2_H_2_ and H_2_ as the source gases and SF_6_ as the additive gas. The deposition reaction conditions for the formation of the various carbon structures are listed in Table 1. Eight samples were prepared using different flow injection processes and different flow rates of the source gases.

**Figure 1:**
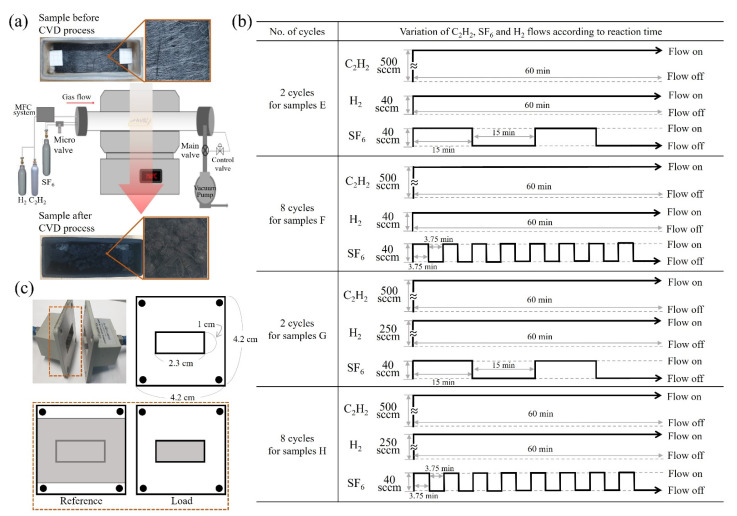
(**a**) Samples before and after thermal chemical vapor deposition (TCVD) process; (**b**) cyclic injection processes of C_2_H_2_, H_2_, and SF_6_ for samples E–H; and (**c**) waveguide test holders for vector network analyzer (VNA).

Figure 1a shows the details of preparation of specimens for the electromagnetic parameters test. CMCs were formed in c-NFs using C_2_H_2_ + SF_6_ gas flow in a thermal chemical vapor deposition system. The incorporation of H_2_ gas flow into the C_2_H_2_ + SF_6_ gas flow system caused the selective hybrid formation of numerous tiny CNFs in the c-NFs, where the CNFs formed only on the surfaces of the i-CFs constituting the c-NFs and not on the surfaces of the CMCs. For samples E–H, cyclic modulation of SF_6_ gas flow was conducted by simply switching the gas flow on and off in continuous cycles. The source gas flow sequence mirrored the iterative order of the reaction processes: C_2_H_2_ + H_2_ + SF_6_ flow (C_2_H_2_ flow on, H_2_ flow on, and SF_6_ flow on) followed by C_2_H_2_ + H_2_ flow (C_2_H_2_ flow on, H_2_ flow on, and SF_6_ flow off), as shown in Figure 1b. The cycle period was defined as the sum of the time the source gases were composed of C_2_H_2_ + H_2_ + SF_6_ flow and the time the source gases consisted solely of C_2_H_2_ + H_2_ flow. For samples E and G, the on and off times for SF_6_ flow injection were set at 15.0 min, resulting in a total duration of 30 min for one cycle. Because the total reaction time was 60 min, a total of two cycles were performed during the reaction. For samples F and H, the on and off times for SF_6_ flow injection were set at 3.75 min. Therefore, the total duration of one cycle was 7.5 min, resulting in a total of eight cycles during the reaction.

The morphologies of the samples were investigated in detail using field-emission scanning electron microscopy (FESEM; S-4200 Hitachi, Tokyo, Japan). Elemental analysis of the samples was performed using energy dispersive X-ray spectroscopy (EDS) in SEM mode at a resolution of 127 eV using an X-Max^N^ SDD detector 51-xmx1010 (Oxford Instruments NanoAnalysis, High Wycombe, UK), and the thickness of the samples was measured using a micrometer (406-250-30 Mitutoyo, Nakagawa, Japan). Resistivity values were obtained using a four-point probe (labsysstc-400 Nextron, Busan, Korea) connected to a source meter (2400 Source Meter, Cleveland, OH, USA) and by performing calculations using Ohm’s law with a correction factor according to the method proposed by Smits [14]. The four-point probe system consisted of four straight-lined probes with equal inter-probe spacings of 3.0 mm. A constant current (I) was supplied through the two outer probes, and the output voltage (V) was measured using the two inner probes [15]. Furthermore, the correction factors (*C* and *F*) were obtained from Smits et al. [14]. Surface and volume resistivities were calculated using the following equations [14,15]:

(1) $$\mathrm{Surface}\;\mathrm{resistivity}:\;\rho_{s}\;=\frac{V}{I}C\left(\frac{a}{d},\frac{d}{s}\right)$$

(2) $$\mathrm{Volume}\;\mathrm{resistivity}:\;{\rho_{v}}_{\;}=\;\rho_{s}\;w\;F\;\left(\frac{w}{s}\right)$$

where *a*, *d*, *w*, and *s* denote the length, width, and thickness of the sample and the inter-probe spacing, respectively.

**Figure 2:**
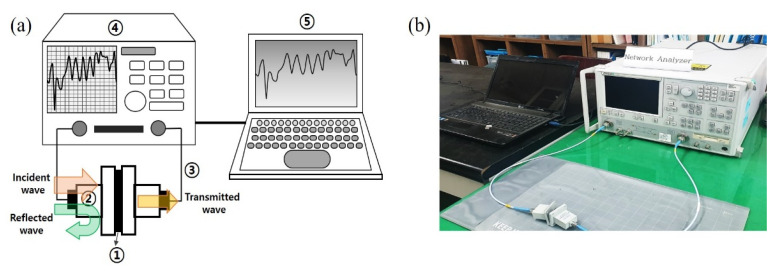
(**a**) Schematic of the vector network analyzer (VNA): ① sample, ② waveguide test holders, ③ coaxial cables, ④ VNA, and ⑤ computer; (**b**) optical photograph of the experimental setup for EMI shielding measurements.

The shielding effectiveness (SE) values of the c-NF samples were measured using the waveguide method with a vector network analyzer (VNA; 37369C, Kanagawa, Japan), as shown in Figure 2. The setup consisted of a sample holder with its exterior connected to the VNA system. A coaxial sample holder and coaxial transmission test specimen were set up according to the waveguide method. The scattering parameters (S_11_ and S_21_) were measured in the 8.0 to 12.0 GHz frequency range using the VNA [16,17,18,19,20]. The power coefficients, namely, reflectivity (R), absorptivity (A), and transmissivity (T), were calculated using the following equations: R = P_R_/P_I_ = |S_11_|^2^ and T = P_T_/P_I_ = |S_21_|^2^, where P_I_, P_R_, P_A_, and P_T_ are the incident, reflected, absorbed, and transmitted powers of an EM wave, respectively [20]. The power coefficient relationships are expressed as R + A + T = 1. The EM wave SE was calculated from the scattering parameters using the following equations:

SE_tot_ = −10log T (3)

SE_R_ = −10log (1 − R) (4)

SE_A_ = −10log{T/(1 − R)} (5)

where SE_tot_, SE_R_, and SE_A_ denote the total, reflection, and absorption SE values, respectively [19,20].

## 3. Results

**Figure 3:**
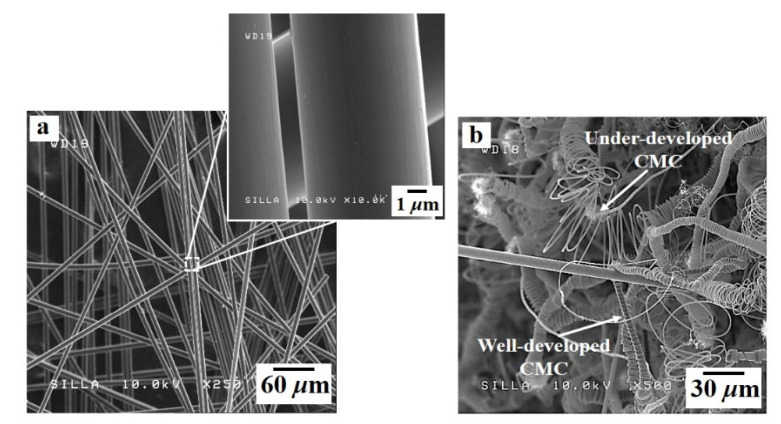
FESEM images of (**a**) sample A and (**b**) sample B. The inset in Figure 3a shows the very clean surfaces of the i-CFs.

**Figure 4:**
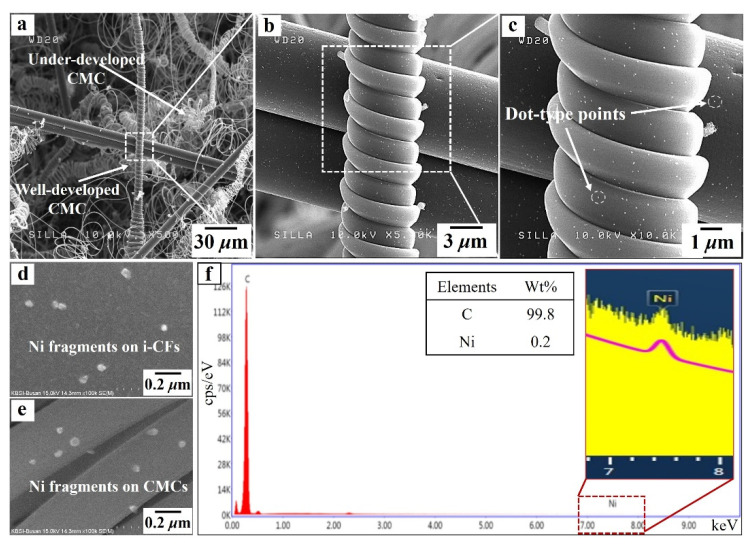
(**a**) FESEM image of sample B, (**b**) magnified FESEM image of Figure 4a, (**c**) magnified FESEM image of Figure 3b, (**d**) high magnification FESEM image of the surface of i-CFs, (**e**) high magnification FESEM image of the surface of carbon microcoils (CMCs), and (**f**) energy dispersive X-ray spectroscopy (EDS) histogram of the surface of the CMCs in Figure 4e.

Figure 3 shows FESEM images of samples A and B. The native c-NFs (sample A) contained many intersections of randomly oriented i-CFs (Figure 3a), and the i-CFs constituting the native c-NFs had clean surfaces (inset in Figure 3a). After TCVD processing of native c-NFs using C_2_H_2_ + SF_6_ gas flow (sample B), both well-developed and under-developed CMCs were observed, as shown in Figure 3b. No CNF-related hybrids formed on the surfaces of either the CMCs or i-CFs in sample B. High-magnification images of sample B revealed the existence of a large number of dots (0.05–0.1 μm in diameter) on the surfaces of both the CMCs and i-CFs (see Figure 4c). EDS analysis indicated that the dots were composed of Ni (see the Ni fragments in Figure 4d,e and the EDS histogram for CMC in Figure 4f). These Ni dots on the surfaces of both the CMCs and i-CFs are thought to be pulverized bunch-type Ni catalysts [21]. The large bunch-type Ni catalysts (100–200 μm in diameter) were broken into a large number of fragments (dots, 0.05–0.1 μm in diameter) during the reaction. Consequently, they were occasionally present on the surfaces of both the CMCs and i-CFs.

**Figure 5:**
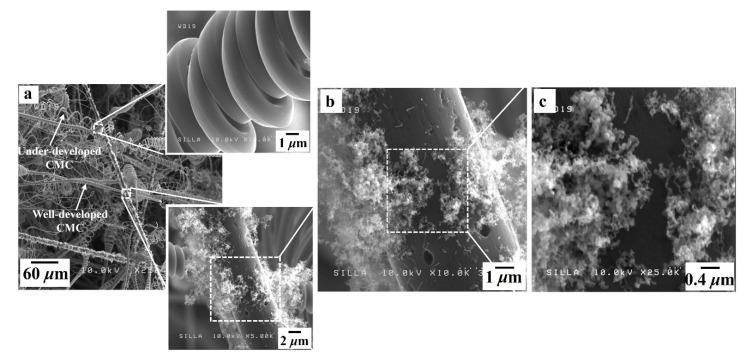
(**a**) FESEM images of sample C, (**b**) magnified FESEM image of the lower inset in Figure 5a, and (**c**) high magnification FESEM image of Figure 5b. The upper and lower insets in Figure 5a show the surfaces of CMCs and i-CFs, respectively.

After the incorporation of H_2_ gas flow into in the C_2_H_2_ + SF_6_ gas flow system (sample C), both well-developed and under-developed CMCs were observed, as shown in Figure 5a. The i-CFs in sample C had shag-shaped materials sporadically arranged on their surfaces (see the lower inset in Figure 5a). High-magnification images of sample C indicated that the shag-shaped materials were composed of numerous tiny CNFs, as shown in Figure 5b,c. Similar to a previous report [21], the hydrogen environment during the reaction produced smaller Ni fragments (less than 0.05 μm in diameter), resulting in the formation of tiny CNFs. Owing to the hydrogen injection process, smaller Ni fragments continuously formed during the reaction and frequently attached to the surfaces of i-CFs. It is suggested that these smaller Ni fragments caused the formation of numerous tiny CNFs along the i-CFs, as shown in the FESEM image of sample C (see Figure 5b) [21].

**Figure 6:**
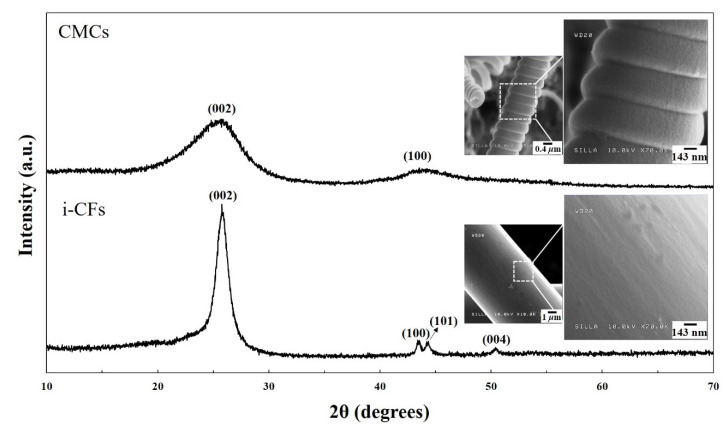
XRD patterns and FESEM images of the CMC and i-CF surfaces.

Interestingly, the shag-shaped materials were not observed on the surfaces of the CMCs; they only formed on the surfaces of the i-CFs (compare the upper and lower insets in Figure 5a). This selective formation of tiny CNFs only on the surfaces of i-CFs and not on the surfaces of CMCs is understood as follows. Previously, producing a hybrid nanocarbon formation (such as CNTs with CNFs and CMCs with carbon nanocoils) was very difficult because the transition metals used as catalysts for nanocarbon growth could easily diffuse into the interior of the carbon substrate during the reaction [22,23]. In this work, the different intrinsic material characteristics of the different carbon-based substrates (namely, CMCs and i-CFs) appears to have directly affected the selective formation of tiny CNFs. As indicated by the X-ray diffraction (XRD) patterns of i-CFs and CMCs in Figure 6, the i-CFs constituting the c-NFs had solid crystalline characteristics. In contrast, the CMCs exhibited an amorphous solid state [24,25,26]. These results reveal that the surfaces of the i-CFs had a compact binding state, while those of the CMCs had a loose binding state. Therefore, compared with those of the i-CFs, the surfaces of the CMCs could be easily penetrated by other materials. Consequently, the smaller Ni fragments on the surfaces of the CMCs (relative to those on the surfaces of the i-CFs) could easily diffuse into the interior of the CMCs. The nearly clean surface state, that is, without any dots, of the CMCs (see the upper inset in Figure 5a) confirms the easier diffusion of smaller Ni fragments from the surfaces of the CMCs into the interior of the CMCs during the CMC formation reaction. Consequently, tiny CNFs did not form on the surfaces of the CMCs because of the lack of smaller Ni catalysts on their surfaces. In contrast, numerous tiny CNFs formed on the surfaces of the i-CFs owing to the compact binding state of their surfaces.

**Figure 7:**
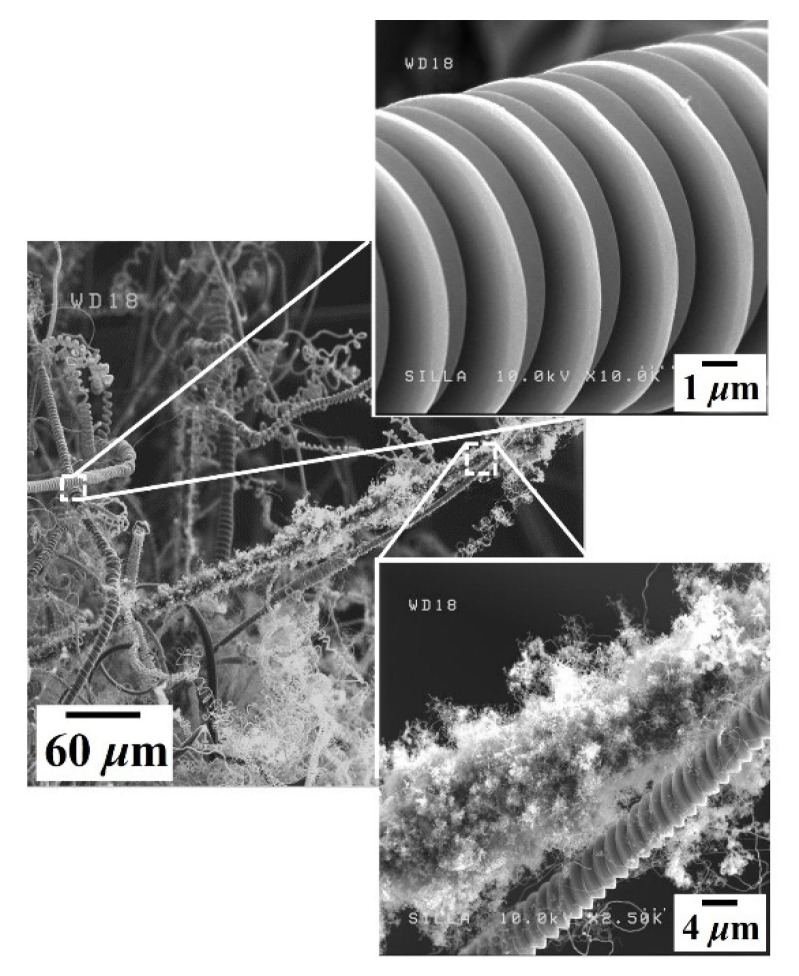
FESEM images of sample D.

When the H_2_ gas flow rate increased from 40 to 250 sccm in the C_2_H_2_ + SF_6_ + H_2_ gas flow system (sample D), the selective formation of numerous tiny CNFs only on the i-CFs surfaces was observed more clearly, as shown in the lower inset in Figure 7. This result strongly confirms that the selective formation of numerous tiny CNFs only on the i-CFs surfaces was due to the incorporation of H_2_ gas flow into the C_2_H_2_ + SF_6_ gas flow system. The nearly clean surface state, that is, without any dots and/or tiny CNFs, of the CMCs in sample D also clearly confirms the easier diffusion of the smaller Ni fragments on the surfaces of the CMCs into the interior of the CMCs (see the upper inset in Figure 7).

**Figure 8:**
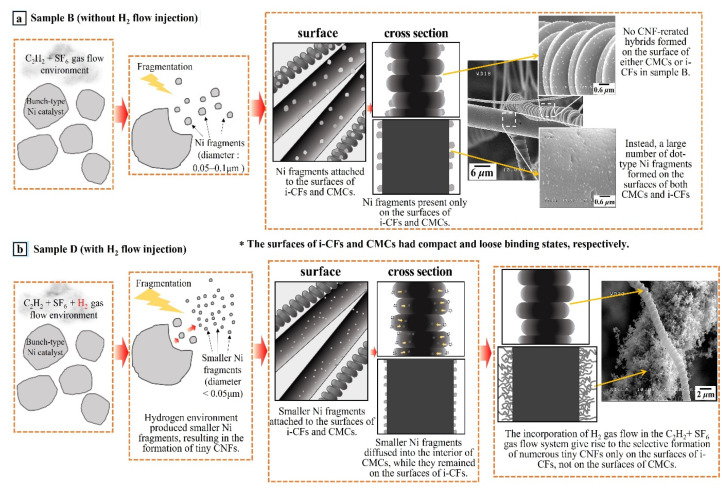
Systematic diagrams indicating (**a**) the existence of a large number of dot-shaped Ni fragments on the surfaces of both CMCs and i-CFs in the C_2_H_2_ + SF_6_ gas flow system and (**b**) the selective formation of numerous tiny CNFs only on the surfaces of i-CFs and not on the surfaces of CMCs due to the incorporation of H_2_ gas flow into the C_2_H_2_ + SF_6_ gas flow system.

Figure 8 shows systematic diagrams for the formation of sample B (C_2_H_2_ + SF_6_ gas flow system, Figure 8a) and sample D (C_2_H_2_ + SF_6_ + H_2_ gas flow system, Figure 8b). In Figure 8a, a large number of Ni fragments are present on the surfaces of both the CMCs and i-CFs. Figure 8b displays the selective formation of numerous tiny CNFs only on the surfaces of i-CFs and not on the surfaces of CMCs.

**Figure 9:**
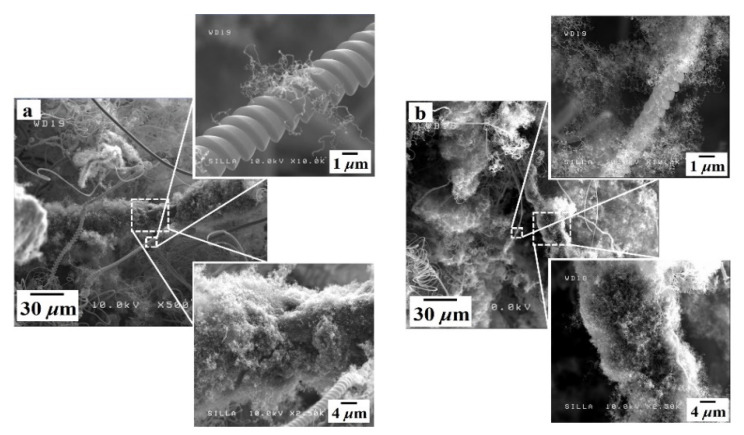
FESEM images of samples (**a**) G and (**b**) H.

Figure 9 shows FESEM images of samples G and H, which were fabricated using cyclic processes. Unlike the surfaces in sample D, numerous tiny CNFs were present on the surfaces of both the CMCs and i-CFs in samples G and H (compare the upper and lower insets in Figure 9 with those in Figure 7). This suggests that the cyclic process noticeably promotes the nonselective formation of numerous tiny CNFs on the surfaces of both i-CFs and CMCs.

Furthermore, Figure 9 shows FESEM images of samples fabricated using cyclic processes with different on/off times for SF_6_ flow: sample G with 15/15 min on/off times for SF_6_ flow (total of two cycles) and sample H with 3.75/3.75 min on/off times for SF_6_ flow (total of eight cycles). As the number of cycles increased from two to eight, the number of tiny CNFs on the surface of the CMCs appeared to increase (compare the upper inset in Figure 9a with that in Figure 9b), indicating that increasing the number of on/off cycles for SF_6_ flow promotes the formation of tiny CNFs.

The nonselective formation of tiny CNFs due to the cyclic process can be explained as follows. The injection of H_2_ flow into C_2_H_2_ flow and an abundance of C_2_H_2_ gas compared with SF_6_ gas in the reaction environment are known to facilitate the formation of numerous tiny CNFs via the generation of smaller Ni catalysts from the large bunch-type Ni catalysts [21,27]. In this work, the H_2_ flow-injected samples without the cyclic process (samples C and D) were subjected to the injection of H_2_ flow into C_2_H_2_ flow, while the H_2_ flow-injected samples with the cyclic process (samples E–H) were subjected to both the injection of H_2_ flow into C_2_H_2_ flow and an abundant C_2_H_2_ gas environment. Consequently, compared with samples C and D, samples E–H produced a greater number of smaller Ni catalysts, which were frequently placed on the surfaces of the CMCs and i-CFs.

Furthermore, a longer period of abundant C_2_H_2_ flow would induce the diffusion of smaller Ni catalysts on the surfaces of CMCs into the interior [5,6,27,28]. Therefore, a shorter period of abundant C_2_H_2_ flow could prevent the diffusion of smaller Ni catalysts into the interior of CMCs and maintain the Ni catalysts on the surfaces of the CMCs, resulting in the formation of numerous tiny CNFs on the surfaces of the CMCs. A higher number of SF_6_ on/off cycles over a fixed period would result in shorter periods of C_2_H_2_ flow. Consequently, it would result in the formation of much more tiny CNFs on the surfaces of the CMCs owing to the larger number of smaller Ni catalysts on their surfaces.

**Figure 10:**
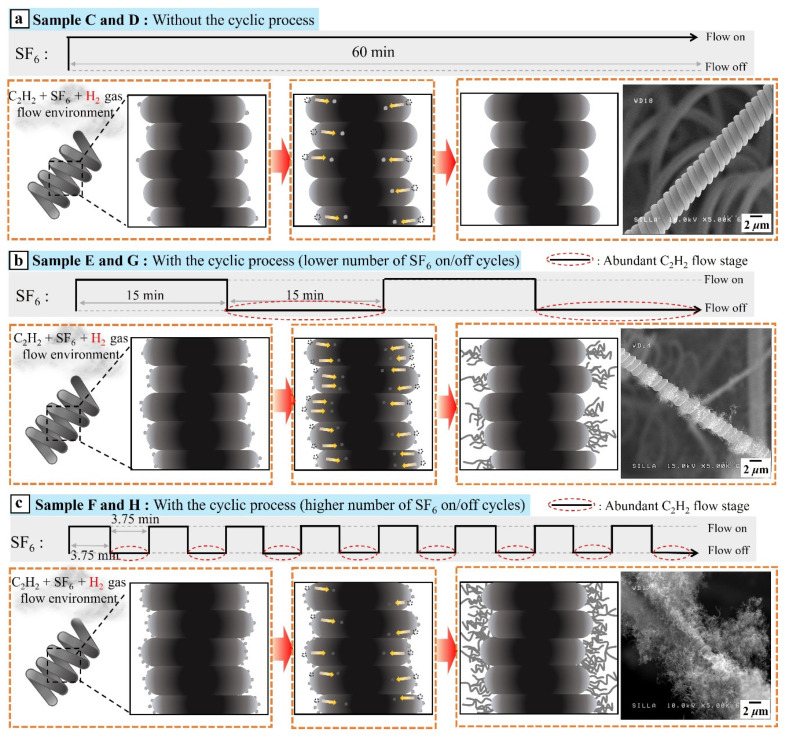
Systematic diagrams of the C_2_H_2_ + SF_6_ + H_2_ gas flow system revealing (**a**) the selective formation of the numerous tiny CNFs only on i-CFs and not on CMCs without the cyclic process, (**b**) the nonselective formation of numerous tiny CNFs caused by the cyclic process, and (**c**) the increase in the number of tiny CNFs on CMCs with increasing frequency of on/off SF_6_ flow cycles.

Figure 10 shows systematic diagrams revealing both the nonselective formation of numerous tiny CNFs caused by the cyclic process and the increase in the number of tiny CNFs caused by the shorter period of abundant C_2_H_2_ flow in the cyclic process.

Although the accurate thickness of each sample could not be precisely measured because of the intrinsic shape of the c-NFs, it was estimated using a micrometer. The thickness of the native c-NFs (sample A) was approximately 1.5 (±0.05) mm. After CMCs formation on the native c-NFs (sample B), the sample thickness increased to 1.8 (±0.35) mm. After hybrid formation (samples D–H), the sample thicknesses were between 1.7 (±0.11) and 4.6 (±0.35) mm.

In addition, the resistivity of each sample was estimated using a four-point probe and the measured sample thicknesses (see Table 2). Although the values measured by the four-point probe could not reveal the exact electrical conductivity values of the samples, the electrical conductivity of the c-NFs appeared to decrease slightly after hybrid formation, as shown in Table 2. This reduction appeared to be due to the formation of superfluous carbon materials in inter-CF spaces or on the surfaces of i-CFs in the c-NFs, as previously reported [6].

To investigate the enhancement in the SE of c-NFs due to hybrid formation, the SE values for the samples were measured in the X-band region (8.0–12.0 GHz). All the samples had total SE values above 40 dB throughout the entire range of operating frequencies. Compared with previously reported total SE values, the presently measured values rank in the top tier (Table 3). Therefore, we suggest that c-NF-based materials, regardless of hybrid formation, can be effectively used in diverse industrial fields.

**Figure 11:**
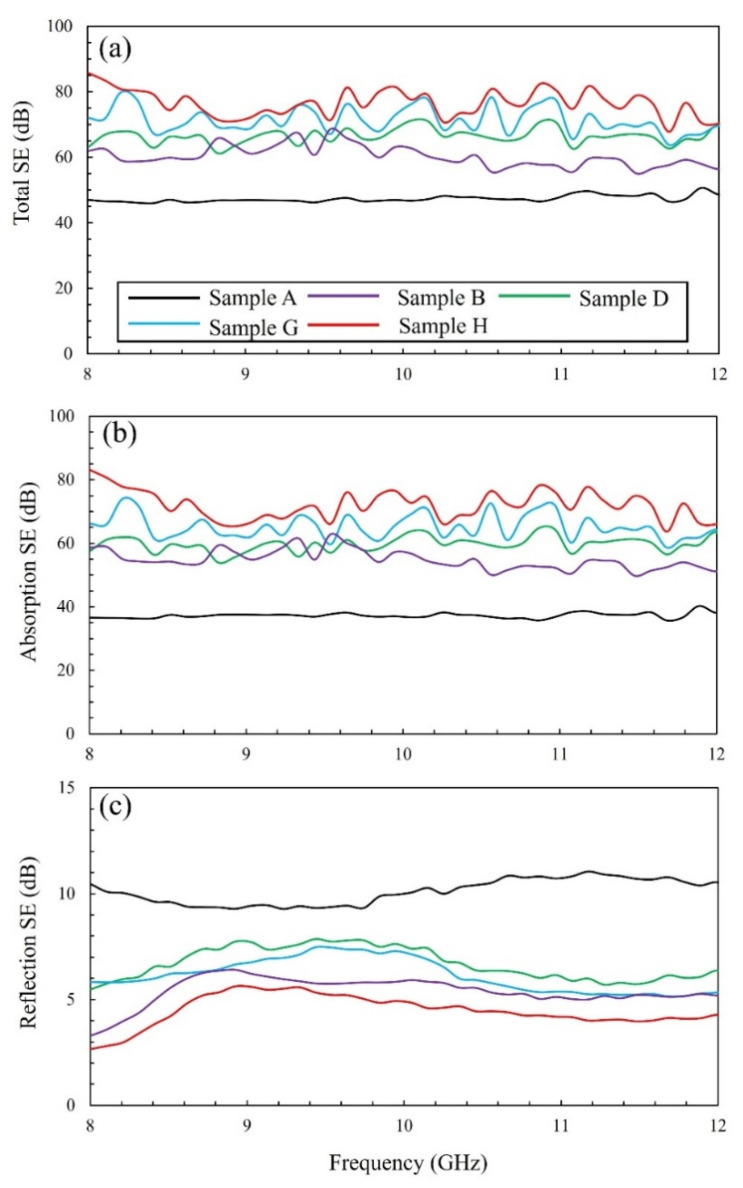
(**a**) Total SE spectra, (**b**) absorption component of the SE spectra, and (**c**) reflection component of the SE spectra for samples A, B, D, G, and H.

As shown in Figure 11a, the total SE values for native c-NFs increased following hybrid formation, although the electrical conductivity of native c-NFs was slightly reduced by the hybridization reaction (Table 2, from 2.17 (±0.07) × 10^3^ S/m for the native c-NFs to 2.16 (±0.46) × 10^3^–4.96 (±0.35) × 10^2^ S/m for the hybridized c-NFs). The improvement in the total SE values according to the deterioration in the electrical conductivity can be explained as follows. The total EMI SE values for electrically conducting materials can be estimated using the empirical equation introduced by Simon [4]:

SE = 50 + 10log_10_(*ρf*)^−1^ + 1.7 *t*(*f*/*ρ*)^1/2^ (6)

where SE is reported in dB, *ρ* is the resistivity at room temperature (Ω·cm), *t* is the thickness of the sample (cm), and *f* is the operating frequency (dB). At high operating frequencies, the empirical equation of Simon can be simplified to SE ∝ 1.7 *t*(*f*/*ρ*)^1/2^. This reveals that the total SE value is directly proportional to the thickness and inversely proportional to the square root of the electrical resistivity of the shielding material. The thickness of the native c-NFs increased significantly following hybrid formation (Table 2, from 1.5 (±0.05) mm for the native c-NFs to 1.7 (±0.11)–4.6 (±0.35) mm for the hybridized c-NFs). Thus, the thicker hybridized c-NFs are expected to provide greater SE. Furthermore, the numerous tiny CNFs in the hybridized c-NFs intersected one another. When an incoming EM wave reaches these fibers, electric current flows into the intersections and drains in various directions, thereby inducing an electromotive force and generating a variable magnetic field [8,9]. The geometry of the c-NFs holds and rotates incoming EM waves within the generated variable magnetic field. Thus, incoming EM wave energy is absorbed into c-NFs and is finally converted into thermal energy [10]. Therefore, these intersections may contribute to the absorption mechanism for the shielding of EM waves. Sample H exhibited the nonselective formation of numerous tiny CNFs on the surfaces of both i-CFs and CMCs, which would produce to the highest number of intersections of all the samples in this study. Consequently, the total SE values of sample H should be higher than those of samples A, B, D, and G. This matches the measured SE values, as shown in Figure 11a. The skin depth (*δ*) of a shielding material is defined as *δ* = (*πσfμ)*^−1/2^ [2], indicating that δ^2^ is inversely proportional to the electrical conductivity (*σ*), frequency (*f*), and magnetic permeability (*μ*). Therefore, a higher magnetic permeability can efficiently reduce the skin depth of the shielding material, thereby enhancing the SE values. The intrinsic characteristics of CMCs in the hybridized c-NFs can generate a magnetic field and absorb incoming EM waves. Consequently, they can enhance the magnetic permeability (*μ*), resulting in an improvement in the absorption loss of EM waves at high operating frequencies. Therefore, the absorption SE values of c-NFs increased following CMC hybrid formation despite the reduction in electrical conductivity.

From sample A to H, both the total SE values and the absorption SE values of the hybridized c-NFs increased for all operating frequencies, as shown in Figure 11a,b. This confirms that the higher total SE values of the hybridized c-NFs in this work are mainly attributable to the enhanced absorption loss at all operating frequencies.

Figure 11c shows the SE values for the reflection loss of the native and hybridized c-NFs. For the reflection loss, the SE values of the hybridized c-NFs were lower than that of the native c-NFs throughout the entire range of operating frequencies. At lowest operating frequency (8.0 GHz) in this work, the difference between the SE values of sample A, with the highest electrical conductivity, and sample H, with the lowest electrical conductivity value, was the largest. The combined results of Table 2 and Figure 11c confirm the dependence of reflection SE of the samples on their electrical conductivity values.

**Figure 12:**
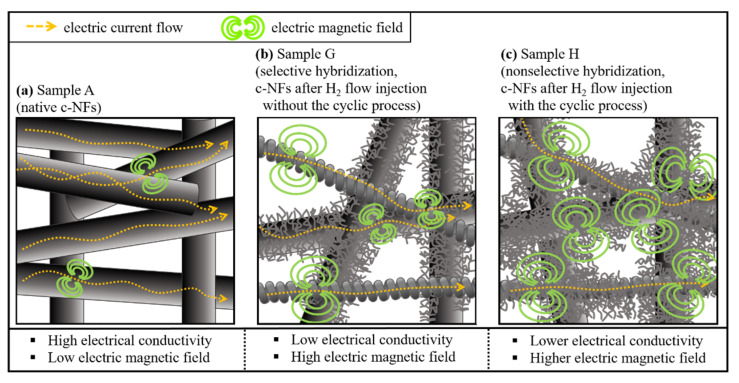
Schematic explaining the enhancement in total SE for the nonselective hybrid formation of numerous tiny CNFs in c-NFs (sample H).

The schematic in Figure 12 explains the reasons behind the enhancement in total SE for the nonselective hybrid formation of numerous tiny CNFs in c-NFs (sample H). It reveals the improvement in the total SE values for the nonselective hybridized sample owing to the enhanced absorption SE originating from the numerous intersections of tiny CNFs, although hybrid formation reduced the electrical conductivity of the sample.

Apart from its excellent SE values, the hybridized c-NFs fabricated in this study could give remarkable mechanical strength when they were manufactured as a stacking type, namely woven-nonwoven-woven type, fabrics. The details for the fabrication of the stacking type fabrics were applied as a Korean patent (application number 10-2020-0108268) [40]. In addition, its production process may be scaled up in a cost-effective manner. These results strongly suggest that the hybridized c-NFs fabricated in this study are applicable for the manufacture of effective and highly flexible EM shielding materials.

## 4. Conclusions

CMCs were formed in c-NFs under C_2_H_2_ + SF_6_ gas flow in a thermal chemical vapor deposition system. The incorporation of H_2_ gas flow into the C_2_H_2_ + SF_6_ gas flow system resulted in the selective hybrid formation of numerous tiny CNFs only on the surfaces of the i-CFs in the c-NFs, not on the surfaces of the CMCs. This selective formation of tiny CNFs is ascribed to the different intrinsic material characteristics of the CMCs and i-CFs. Systematic diagrams are presented that explain how the incorporation of H_2_ gas flow into the C_2_H_2_ + SF_6_ gas flow system causes the selective hybrid formation of numerous tiny CNFs.

The nonselective hybrid formation of numerous tiny CNFs on the surfaces of both the i-CFs and CMCs in c-NFs was achieved using a cyclic process in the C_2_H_2_ + SF_6_ + H_2_ gas flow system. The production of a larger number of smaller Ni catalysts caused by the abundant C_2_H_2_ gas environment during the reaction appeared to be the main cause for this nonselective hybrid formation. Furthermore, increasing the number of SF_6_ on/off cycles in the cyclic process increased the number of tiny CNFs that formed on the surfaces of the CMCs because it maintained the smaller Ni catalysts on the CMC surfaces. Systematic diagrams are presented that explain both the nonselective formation of numerous tiny CNFs by the cyclic process and the increase in the number of tiny CNFs on the surfaces of the CMCs with increasing number of SF_6_ on/off cycles in the cyclic process.

Although hybrid formation reduced the electrical conductivity of native c-NFs, the total SE values for native c-NFs greatly increased following hybrid formation across operating frequencies in the 8.0–12.0 GHz range. This dramatic improvement in the total SE values is ascribed to the enhanced absorption SE originating from the intrinsic characteristics of the CMCs, the numerous intersections of tiny CNFs, and the increased thickness of the c-NFs after hybrid formation.

## Figures and Tables

**Table 1:** Experimental conditions for samples A–H.

Samples	C_2_H_2_ Gas Flow Rate (sccm)	H_2_ Gas Flow Rate (sccm)	SF_6_ Gas Flow Rate (sccm)	No. of SF_6_ Cycles On/Off	Total Gas Pressure (Torr)	Total Reaction Time (min)	Substrate Temp. (°C)	Remarks
A	-	-	-	-	-	-	-	Native c-NFs
B	500	-	40	-	100	60	750	Without cyclic process
C	500	40	40	-	100	60	750	Without cyclic process
D	500	250	40	-	100	60	750	Without cyclic process
E	500	40	40	2	100	60	750	With cyclic process
F	500	40	40	8	100	60	750	With cyclic process
G	500	250	40	2	100	60	750	With cyclic process
H	500	250	40	8	100	60	750	With cyclic process

**Table 2:** Thickness, volume resistivity, and electrical conductivity of samples A, B, D, G, and H.

Samples	Thickness *t* (mm)	Electrical Resistivity *ρ* (Ω·m)	Electrical Conductivity *σ* (S/m)	* Correction Factor *F* (F/w)
A	1.5 (±0.05)	4.61 (±0.14) × 10^−4^	2.17 (±0.07) × 10^3^	0.99
B	1.8 (±0.35)	4.64 (±1.23) × 10^−4^	2.16 (±0.46) × 10^3^	0.98
D	1.7 (±0.11)	4.92 (±0.65) × 10^−4^	2.03 (±0.23) × 10^3^	0.98
G	2.9 (±0.78)	1.07 (±0.29) × 10^−3^	9.33 (±1.98) × 10^2^	0.92
H	4.6 (±0.35)	2.02 (±0.15) × 10^−3^	4.96 (±0.35) × 10^2^	0.79

* The correction factor was calculated from Table 3 in Ref. [14].

**Table 3:** EMI SE of carbon-based materials.

Carbon-Based Materials	Thickness (mm)	Electrical Conductivity or Resistivity	Operating Frequency (GHz)	SE (dB)	Ref.
15 wt% * CB/* ABS	1.1	-	8.2–12.4	21	[29]
15 wt% * CNF/* ABS		1.5 ± 0.1 Ω·cm		35	
15 wt% * CNT/* ABS		0.81 ± 0.05 Ω·cm		51	
* CNF/epoxy	2.1	-		5–34	[30]
* CNT macro-films	0.004	-		61–67	[31]
* SCF/* EVA	3.5	-	8–12	29.5–34.1	[32]
* MX/* RGO	3	1000 S/m		51	[33]
* 3D G–CNT–Fe_2_O_3_	0.6	22,781 S/m		130–134	[34]
* GN/Cu	0.009 (±0.0015)	5.88 (±0.29) × 10^6^ S/m	1–18	52–63	[35]
* THCS/paraffin	2.8	-	2–18	48.5	[36]
* CNTsM	4.6	-	2–18	35	[37]
* M40J SCF felt	0.422	1.88 × 10^3^ S/m	8.2–12.4	66.7–71.4	[38]
* CF-1200	4	0.159 S/cm	8–12	64	[39]
* c-NFs	1.5 (±0.05)	2.17 (±0.07) × 10^3^ S/m	8–12	46–53	This work
* CMCs on c-NFs	1.8 (±0.35)	2.16 (±0.46) × 10^3^ S/m		55–68	
* CNF–i-CF hybrids in c-NFs	1.7 (±0.11)	2.03 (±0.23) × 10^3^ S/m		61–75	
* CNF–CMC and CNF–i-CF hybrids in c-NFs	2.9 (±0.78)–4.6 (±0.35)	4.96 (±0.35)–9.33 (±1.98) × 10^2^ S/m		64–86	

* CB: carbon black, * CNF: carbon nanofiber, * CNT: carbon nanotube, * ABS: acrylonitrile–butadiene–styrene, * SCF: short carbon fiber, * EVA: ethylene vinyl acetate, * MX: Mxene, * RGO: reduced graphene oxide, * 3D G–CNT–Fe_2_O_3_: three-dimensional graphene–carbon nanotube–iron oxide, * GN: graphene, * THCS: triple-shell hollow carbon submicrospheres, * CNTsM: carbon nanotubes microspheres, * M40J SCF felt: M40J short-chopped carbon fibers felt, * CF-1200: carbon foams after carbonized at 1200 °C, * c-NFs: native carbon-based nonwoven fabrics, * CMCs on c-NFs: carbon microcoils grown on c-NFs, * CNF–i-CF hybrids on c-NFs: hybrid of tiny CNFs on i-CFs in c-NFs, * CNF–CMC and CNF–i-CF hybrids in c-NFs: hybrids of both tiny CNFs on CMCs and tiny CNFs on i-CFs in c-NFs.

## Data Availability

All data used and/or analyzed during the current study are shown within the study. If further data are required, it can be made available by the corresponding author upon reasonable request.
